# A New Normal: Trends of Upper Extremity Orthopaedic Injuries Nationwide During the COVID-19 Pandemic

**DOI:** 10.7759/cureus.30299

**Published:** 2022-10-14

**Authors:** Alexis B Sandler, Michael D Baird, Steven S Kurapaty, John P Scanaliato, John C Dunn, Nata Parnes

**Affiliations:** 1 Department of Orthopaedic Surgery, William Beaumont Army Medical Center, Texas Tech University Health Sciences Center, El Paso, USA; 2 Department of Orthopedic Surgery, Walter Reed National Military Medical Center, Bethesda, USA; 3 Department of Orthopedic Surgery, Northwestern University Feinberg School of Medicine, Chicago, USA; 4 Department of Orthopedic Surgery, William Beaumont Army Medical Center, Texas Tech University Health Sciences Center, El Paso, USA; 5 Department of Orthopedic Hand Surgery, William Beaumont Army Medical Center, El Paso, USA; 6 Department of Orthopedic Surgery, Claxton-Hepburn Medical Center, Ogdensburg, USA; 7 Department of Orthopedic Surgery and Rehabilitation, Carthage Area Hospital, Carthage, USA

**Keywords:** orthopedics, emergency medical system, upper extremity trauma, epidemiology, covid-19, upper extremity injuries

## Abstract

Background

The widespread societal effects of the COVID-19 pandemic connote public health and epidemiological changes for orthopedic injuries. The epidemiology of upper extremity injuries and the effects of the pandemic on these nationwide trends is poorly defined.

Methods

This cross-sectional, descriptive epidemiological study compares epidemiological trends among upper extremity (UE) orthopedic injuries presenting to emergency departments (EDs) prior to and during the COVID-19 pandemic. Upper extremity fracture and dislocation data was sourced from the National Electronic Injury Surveillance System (NEISS) database in years prior to (2015-2019) and during the pandemic (2020-2021). Data on incidence, patient demographics, injury patterns, mechanisms of injury, incident locale, and patient disposition were collected and compared between years.

Results

The pre-COVID-19 incidence rate (IR) of UE fractures at 2.03 per 1,000 persons (n=3038930 from 2015-2019) decreased to 1.84 per 1,000 in 2020 (n=474805) and 1.82 per 1,000 in 2021 (n=471793). Dislocation rates were largely unchanged at 0.34 per 1,000 people (n=476740) prior to the pandemic and with incidence rates of 0.33 per 1,000 (n=85582) and 0.34 per 1,000 (n=89386) in 2020 and 2021, respectively. Female patients over 65 had the highest injury IR at 4.85 per 1,000 (n=976948). Finger fractures (IR=0.38 per 1000, n=96009) overtook hand fractures (IR=0.51 per 1000, n=310710) as more common during COVID-19 in males, while wrist (IR=0.55 per 1000, n=350650) fractures remained most common in females. Injuries from individual sports, such as skateboarding and bicycling, increased during the pandemic, while injuries from team sports decreased. Hospital admission and observation increased in 2020, while discharge and transfer rates decreased. Admission, observation, and discharge rates moved closer to pre-pandemic levels in 2021.

Conclusions

The COVID-19 pandemic was associated with epidemiological and activity changes regarding UE fractures and dislocations presenting to EDs. The present study demonstrates notable decreases in rates of upper extremity fractures and dislocations, increases in rates of injuries related to outdoor and individual sports such as skateboarding with corresponding decreases in rates of injuries related to organized sports such as basketball, increases in the rates of injuries occurring in homes and in association with pet supplies, and decreases in rates of injuries occurring in schools and places of recreation observed during the pandemic. Additionally, trends observed among patient disposition specific to the pandemic, such as increasing rates of patient admission, observation, and against medical advice (AMA) departure with decreasing rates of discharge and transfer, offer insight into the burden of upper extremity injuries on the healthcare system during this critical time. While upper extremity orthopedic injuries remained common through the pandemic, the early pandemic was associated with higher rates of hospital admission that normalized closer to pre-pandemic levels by 2021, which may herald a shift and return to pre-pandemic trends. Future research will determine the long-term downstream effects of COVID-19 on activity-related orthopedic injuries and bone health.

## Introduction

The novel coronavirus (COVID-19) emerged in China at the end of 2019, rapidly spreading worldwide to reach pandemic status by early 2020 [1]. Almost overnight, COVID-19 dramatically introduced new societal norms regarding work, leisure, and education. The ensuing effects of these vicissitudes are evident throughout orthopedics: the transition to telework and remote operations exacerbated existing musculoskeletal neck and lower back pain [2]. The advent of isolation and home quarantine increased the incidence of fractures, dislocations, and head trauma caused by domestic violence [3,4], and the imposition of shelter-in-place orders correlated with increased volumes of upper extremity injuries necessitating emergency surgery and injuries caused by assault and high-speed auto accidents [5]. 

Despite such massive paradigm shifts in society, there is limited literature discussing orthopedic epidemiology on a national level and the burden of orthopedic injuries on the emergency health system, particularly regarding longer-term trends seen when entering the second year of the pandemic. Prior to COVID-19, the incidence of upper extremity fractures was estimated at 0.28 - 0.72 per 1,000 individuals, while the incidence of upper extremity dislocations was estimated at under 0.27 per 1,000 individuals [6,7]. An Italian study demonstrated globally decreased rates of shoulder and elbow trauma related to low-energy mechanisms, sports injuries, and traffic accidents [8]. However, the incidence of low-energy traumatic mechanisms among elderly patients introduced challenges in managing these patients with hospital wards occupied by COVID-19 patients [8].

Many of the studies discussing the orthopedic implications of COVID-19 are specific to a single institution or region. We aim to identify the national implications of the COVID-19 pandemic on upper extremity orthopedic injuries and to better quantify the burden of these injuries on the emergency health system. Using the National Electronic Injury Surveillance System (NEISS), we will explore the effect of the COVID-19 pandemic on upper extremity fractures and dislocations with the hypothesis that emergency department (ED) visits for these injuries decreased during pandemic years and describe changes in injury presentation, trends in patient disposition, and potential implications for future practice.

## Materials and methods

This study offers descriptive epidemiological insight into the impact of COVID-19 on orthopedic upper extremity injuries presenting to emergency departments (EDs) in the US. Data originates from the National Electronic Injury Surveillance System (NEISS) database, which was established by the US Consumer Product Safety Commission (CSPC) in 1978 to aid governmental agencies in monitoring product-related injuries across the country. Data collection for the NEISS is sourced from approximately 100 EDs that represent a probability sample of the over 5000 EDs across the country, which allows results from the NEISS database to be extrapolated to calculate national estimates of injury rates. On an ED level, individual patient data are collected and deidentified by a designated NEISS hospital coordinator through clinical records and follow-up telephone communication. NEISS data for the previous year are released in April of the following year. 

Database query

The NEISS database was queried for all primary fracture and dislocation injuries occurring in patients 18 years or older between 2015 and 2021 in the following anatomic regions: shoulder, upper arm, elbow, lower arm, wrist, hand, and finger. Pre-COVID-19 years were defined as 2015-2019, while COVID-19 years were defined as 2020 and 2021. 

Data Collection

Estimated national incidences were calculated based on patients presenting in the NEISS system, the NEISS weighted estimates, and existing census data and population projections. Patient demographics include patient age and sex, with patient age categorized as 18-44, 45-64, and over 65 years old. Of note, the NEISS database only included non-binary codes for patient sex as early as January 1st, 2021. Injury patterns were classified as either fractures or dislocations and categorized according to the corresponding body part. The mechanism of injury was collected based on the primary product linked to the injury. Mechanisms of injury were classified as “sports” and “non-sports” related categories for further analysis. The incident locale was defined as the location where the injury took place and included home, farm/ranch, street/highway, other public property, manufactured (mobile) home, industrial place, school, and place of recreation or sports. Patient disposition was coded as treated and released; treated and transferred; treated and admitted; held for observation; left either against medical advice (AMA) or without being seen; or fatality, which includes death prior to ED arrival, death in the ED, and death after admission.

Statistical analysis

Raw and weighted descriptive statistics were calculated for included patients. Incidence rates (IRs) per thousand persons at risk and corresponding 95% confidence intervals (CIs) were also calculated based on US census data population and by demographic-based distribution numbers. All descriptive statistics are reported based on weighted population estimates as well as based on raw NEISS data, with relevant population sample sizes and raw NEISS data sample sizes reported for each. Incidence rate ratios (IRRs) were used to compare pre-pandemic and during-pandemic injury rates as well as injury rates between specific years. IRRs are considered statistically significant if both upper and lower limits of the 95% CI are either greater than or less than one. Student t-tests were used to compare injury rates over consecutive years with significance defined as an alpha of 0.05. 

## Results

Incidence

**Figure 1:**
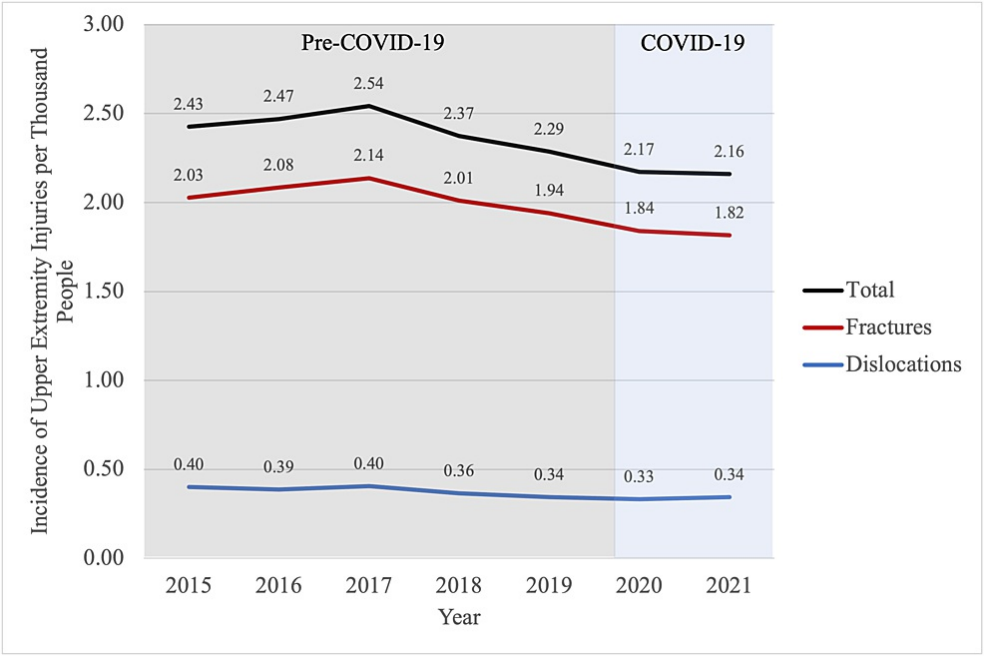
Incidence of upper extremity injuries per thousand people by year

In the years prior to COVID-19, an estimated 2.42 per 1000 people presented to EDs with upper extremity fractures or dislocations (population n=3,038,931, NEISS n=67,354). Injury rates decreased to 2.17 and 2.16 per 1000 people in 2020 and 2021, respectively (population n=560,387, NEISS n=12,839; population n=56,1179, NEISS n=13,581) (Figure 1).

An estimated 2.03 per 1000 people (population n=2,562,191, NEISS n=56,349) presented to EDs with upper extremity fractures while 0.38 per 1000 people (population n=476,740, NEISS data n=11,005) presented with upper extremity dislocations prior to 2020. During COVID-19, these incidences decreased 9.9% and 10.5% (IRR=0.897, CI=0.894-0.899, IR=1.83 per 1000, population n=946,578, NEISS data n=22,196; IRR=0.891, CI=0.886-0.895, IR=0.34 per 1000, population n=174,968, NEISS data n=4224, respectively). There were no significant differences observed in rates of injury between the years 2015 and 2019 (t(8)=0.819, p=.436). Between 2020 to 2021, the incidence of fractures presenting to EDs decreased (IRR=0.987, CI=0.983 - 0.981) while the incidence of dislocations presenting to EDs increased (IRR=1.037, CI=1.027-1.047). 

Demographics

Incidence rates varied significantly between male and female patients in both pre-COVID-19 years (t(8)=2.696, p=.027) and COVID-19 years (t(2)=8.320, p=.014). Proportionally more female patients sustained upper extremity fractures (52.8%, population n= 1,853,086, NEISS n=39,873) while more male patients sustained dislocations (68.0%, population n=10,563, NEISS n=15,229) (Table 1).

**Table 1:** Demographics IR = incidence rate per 1000 persons, NEISS = National Electronic Injury Surveillance System. Please note that non-binary was only added as an option for NEISS sex collection data on January 1st, 2021.

		Fractures				Dislocations			
		IR	Population-based proportion (%)	Population Estimate (n)	NEISS data (n)	IR	Population-based proportion (%)	Population Estimate (n)	NEISS data (n)
Total									
Pre-COVID-19		2.04	84.3%	2,562,191	56,349	0.38	15.7%	476,740	11,005
COVID-19		1.83	84.4%	946,598	22,196	0.34	15.6%	174,968	4224
Age									
Pre-COVID-19	18-44	1.74	33.4%	1,014,181	23,650	0.48	9.3%	282,516	6862
	45-64	1.63	22.5%	682,810	15,095	0.25	3.4%	104,759	2302
	65+	3.41	28.5%	865,200	17,604	0.35	2.9%	89,466	1841
COVID-19	18-44	1.49	31.7%	355,848	8992	0.41	8.7%	97,335	2499
	45-64	1.46	22.1%	247,464	5802	0.22	3.3%	37,374	868
	65+	3.15	30.6%	343,286	7402	0.37	3.6%	40,259	857
Sex									
Pre-COVID-19	Male	1.98	39.9%	1,211,767	27,852	0.53	10.8%	326,938	7679
	Female	2.10	44.4%	1,350,423	28,497	0.23	4.9%	149,802	3326
COVID-19	Male	1.7	39.6%	443,935	10,820	0.46	10.4%	116,238	2884
	Female	1.9	44.8%	502,662	11,376	0.22	5.2%	58,713	1339
	Nonbinary	N/A	N/A	N/A	0	N/A	N/A	16	1

The highest incidence of fractures and dislocations was observed among females over 65 years (IR=4.85, population n=976,948, NEISS n=20,149) followed by males ages 18-44 (IR=3.04, population n=1,263,629, NEISS n=30,590. In comparing the age groups with the highest incidence of dislocations (18-44 years) and age groups with the highest incidence of fractures (over 65 years), the incidence rate of dislocations among the highest age group (patients ages 18-44) decreased more than the incidence rate of fractures in patients over 65 (IRR=0.841, CI =0835-0.847; IRR=0.925, CI=0.921-0.928, respectively). 

Injury patterns

Prior to COVID-19, fractures in male patients most commonly involved the hand (IR=0.51 per 1000, population n=310,710, NEISS n=7127), finger (IR=0.30 per 1000, population n=231,572, NEISS data n=5405), or shoulder (IR=0.30 per 1000, population n=158,817, NEISS data n=4136) (Table 2).

**Table 2:** Injury patterns F = female, M = male, IR = incidence rate per 1000 persons, NEISS = National Electronic Injury Surveillance System

		Pre-COVID-19			COVID-19		
		IR	Population estimate (n)	NEISS data (n)	IR	Population estimate (n)	NEISS data (n)
Fractures							
Finger	M	0.38	231,572	5405	0.38	96,009	2297
	F	0.25	160,783	3608	0.19	50,204	1229
Hand	M	0.51	310,710	7127	0.36	91,535	2307
	F	0.16	102,417	2227	0.12	31,524	762
Wrist	M	0.29	180,415	4292	0.27	68,367	1754
	F	0.55	350,650	7680	0.52	137,174	3195
Lower arm	M	0.17	105,223	2287	0.16	39,421	916
	F	0.30	191,806	3662	0.29	75,729	1535
Elbow	M	0.11	64,648	1576	0.12	29,401	747
	F	0.14	90,161	1875	0.12	31,841	764
Upper arm	M	0.15	92,808	2132	0.16	41,886	977
	F	0.45	230,472	4843	0.42	111,059	2516
Shoulder	M	0.30	185,817	4136	0.30	77,317	1822
	F	0.25	162,094	3317	0.25	65,131	1375
Dislocations							
Finger	M	0.12	77,918	1828	0.19	24,641	606
	F	0.044	28,912	621	0.074	9801	204
Hand	M	0.0059	3629	102	0.013	1678	41
	F	0.0011	686	15	0.0029	385	9
Wrist	M	0.0041	2527	71	0.0082	1040	30
	F	0.0029	1894	37	0.0040	530	10
Lower arm	M	0.00062	386	7	0.00026	33	2
	F	0.00052	335	6	0.0011	145	4
Elbow	M	0.023	14,257	360	0.043	5522	142
	F	0.023	14,955	343	0.055	7276	169
Upper arm	M	0.0014	875	25	0.00051	65	4
	F	0.00038	244	8	0.00092	122	4
Shoulder	M	0.37	227,346	5286	0.33	83,259	2059
	F	0.16	102,776	2296	0.15	40,455	939

During COVID-19, finger fractures emerged as more common than hand fractures in males (finger: IR=0.38 per 1000, population n=96,009, NEISS n=2297; hand: IR=0.36 per 1000, population n=91,535, NEISS n=2307). Hand fractures in males were observed at 0.710 (CI=0.704-0.715), the rate of the prior incidence during COVID-19, while finger fractures were not correlated with a significant change in IRR (IRR=0.999, CI=0.991-1.006). Both prior to and during COVID-19, fractures in female patients most frequently involved the wrist (IR=0.55 per 1000, population n=350,650, NEISS n=7680; IR=0.52 per 1000, population n=137,174, NEISS n=3195), upper arm (IR=0.45 per 1000, population n=230,472, NEISS data n=4843; IR=0.42 per 1000, population n=111,059, NEISS data n=977), or lower arm (IR=0.30 per 1000, population n=191,806, NEISS data n=3662; IR=0.29 per 1000, population n=75,729, NEISS data n=1535). Wrist fractures in females were observed at 0.955 (CI=0.949-0.960) of the prior incidence during COVID-19. Shoulder dislocations were the most common dislocation across all patient sexes (2015-2019: male IR=0.372, population n=227,346, NEISS n=5286, female IR=0.0160, population n=102,776, NEISS n=2296; 2020-2021: male IR=0.378, population n=83,259, NEISS n=2059, female IR=0.153, population n=40,455, NEISS n=939). 

Mechanism of injury 

Table 3 presents the top ten non-sports-related and sports-related products associated with upper extremity injuries before and during the pandemic and corresponding IRRs.

**Table 3:** Associated products ATV = all-terrain vehicle, CI = 95% confidence interval, ED = emergency department, IR = incidence rate per 1000 persons, IRR = incidence rate ratio compared to pre-COVID-19 years, SD = standard deviation

	% of patients presenting to the ED	IR		IRR	SD	CI
		Pre-COVID-19	COVID-19			
Non-sports						
Floors or flooring materials	10.3%	0.25	0.22	0.88	0.0034	0.87-0.89
Stairs or steps	10.2%	0.25	0.22	0.87	0.0035	0.86-0.88
Ceilings or walls	5.7%	0.14	0.11	0.74	0.0049	0.74-0.75
Beds or bedframes	4.2%	0.10	0.097	0.96	0.0053	0.95-0.97
Doors	2.8%	0.071	0.054	0.76	0.0069	0.75-0.77
Ladders	2.7%	0.065	0.057	0.87	0.0068	0.86-0.88
Chairs	2.0%	0.050	0.042	0.83	0.0079	0.81-0.84
Bathtubs or showers	1.8%	0.045	0.039	0.87	0.0082	0.86-0.89
Pet supplies	1.5%	0.035	0.037	1.08	0.0086	1.06-1.09
Porches/balconies	1.3%	0.033	0.028	0.84	0.0097	0.82-0.85
Sports						
Bicycles	6.0%	0.14	0.144	0.99	0.0044	0.98-0.99
Basketball	2.8%	0.074	0.042	0.57	0.0075	0.56-0.58
Skateboards	1.9%	0.039	0.055	1.39	0.0075	1.37-1.41
Exercise without equipment	1.8%	0.040	0.046	1.14	0.0079	1.12-1.16
Football	1.5%	0.041	0.023	0.57	0.010	0.56-0.59
ATV	1.4%	0.033	0.032	0.95	0.0092	0.93-0.97
Dirt Bikes	0.97%	0.022	0.024	1.05	0.011	1.03-1.07
Soccer	0.93%	0.025	0.015	0.61	0.013	0.60-0.63
Roller skating	0.79%	0.018	0.019	1.00	0.012	0.98-1.03
Horseback riding	0.69%	0.016	0.016	0.97	0.013	0.95-1.00

Floors and flooring materials were the most common non-sports-related product associated with orthopaedic upper extremity injuries, most frequently causing upper arm fractures both prior to and during COVID-19 (IRR=0.926, CI=0.914-0.938; pre-COVID-19: IR=0.07 per 1000, population n=83,877, NEISS n=1699; COVID-19: IR=0.06 per 1000, population n=31,996, NEISS n=717). Bicycles were the most common sports-related product associated with injuries and most commonly were associated with shoulder fractures (IRR=0.956, CI=0.940-0.971; pre-COVID-19: IR=0.04 per 1000, population n=53,217, NEISS n=1250; COVID-19: IR=0.04 per 1000, population n=20,957, NEISS n=521). Compared to years prior to COVID-19, the incidence of bicycle-related injuries increased in 2020 but decreased in 2021 (IRR=1.068, CI=1.057-1.080; IRR=0.905, CI = 0.894-0.916). Of the top ten products, incidence rates of injuries related to pet supplies and skateboarding increased (IRR=1.076, CI=1.058-1.094; IRR=1.392, CI=1.372-1.413). Incidence rate ratios of injuries related to pet supplies increased more in 2021 than in 2020 (IRR=1.076, CI=1.091-1.139), while rates of injuries related to skateboarding increased more in 2020 than in 2021 (IRR=1.293, CI=1.268-1.318; IRR=1.492, CI=1.465-1.520). Of all sports-related injuries, rates of basketball injuries decreased the most (IRR=0.571, CI=0.563-0.580).

Incident locale

Incidence of upper extremity fractures and dislocations occurring in the home was 1.12 per 1000 (population n=1,408,177, NEISS n=29,435) prior to COVID-19, with a decreased incidence observed in 2020 (IRR=0.87, CI=0.87-0.88) and more markedly decreased incidence observed in 2021 (IRR=0.80, CI=0.79-0.80) (Table 1). In addition to the decreased incidence rates, proportions of upper extremity injuries occurring in homes also decreased (pre-COVID-19: 46.3%, n=1,408,176 / 3,038,931; COVID-19: 43.3%, n=485,108 / 1,121,566). The incidence of upper extremity fractures and dislocations occurring in schools was 0.014 per 1000 prior to 2020 (population n=17,537, NEISS n=353) and decreased substantially in 2020 (IRR=0.387, CI=0.367-0.409) but less in 2021 (IRR=0.564, CI=0.539-0.590). Similarly, injuries occurring in places of recreation and sports decreased from 0.29 per 1000 (population n=366,888, NEISS n=8762) to 0.20 in 2020 (IRR=0.694, CI=0.688-0.701) before increasing to 0.26 in 2021 (IRR=0.891, CI=0.883-0.898). The incidence of upper extremity injuries on farms and ranches more than doubled in 2020 (IRR=2.129, CI=1.904-2.381) and even quadrupled in 2021 (IRR=4.151, CI=3.807-4.526). 

Patient disposition

Rates of admission, observation, and AMA departures increased during the COVID-19 pandemic, while rates of discharge from the ED and hospital transfer both decreased (Table 4).

**Table 4:** Patient disposition AMA = against medical advice, CI = 95% confidence interval, ED = emergency department, IRR = incidence rate ratio compared to pre-COVID-19 years, SD = standard deviation

	% of patients presenting to the ED	2020			2021		
		IRR	SD	CI	IRR	SD	CI
Discharged	88.6%	0.869	0.00156	0.8670-0.872	0.878	0.00155	0.875-0.880
Admitted	9.1%	1.161	0.00446	1.151-1.172	1.014	0.00470	1.004-1.023
Transferred	1.2%	0.863	0.0133	0.841-0.886	0.871	0.0132	0.849-0.894
Held for observation	0.78%	1.348	0.0146	1.310-1.387	1.023	0.0163	0.991-1.057
Left AMA	0.35%	1.012	0.0250	0.964-1.063	1.811	0.0199	1.741-1.882

Admission and observation rates decreased in 2021 as compared to 2020 (IRR=0.873, CI=0.863-0.883; IRR=0.759, CI=0.730-0.789, respectively) while discharge and AMA departure rates increased (IRR=1.010, CI=1.006-1.014; IRR=1.779, CI=1.692-1.891). Transfer rates did not change significantly between 2020 and 2021 (IRR=1.009, CI=0.976-1.044).

## Discussion

The overall number of upper extremity fractures and dislocations presenting to US EDs decreased substantially in both 2020 and 2021, directly in relation to the rise of the COVID-19 pandemic. Fracture rates were highest among female patients over 65, and dislocation rates were highest among male patients aged 18-44. Finger fractures overtook hand fractures as the most common fracture pattern in male patients, while wrist fractures remained the most common in female patients. The incidence of injuries related to skateboarding increased, with the most drastic decrease observed among basketball injuries. There was an increase in injuries related to pet supplies. Injuries occurring in homes decreased more in 2021 than in 2020, while injuries in schools decreased more in 2020 than in 2021. Additionally, the pandemic engendered increased admission, observation, and AMA departure rates, as well as decreased discharge and transfer rates. Ultimately, the COVID-19 pandemic appears to have altered the epidemiology and ED course of upper extremity orthopedic injuries; however, the transition to more normalized injury patterns in 2021 may herald a shift to pre-pandemic injury rates and patterns as society adapts to COVID-19. 

Early pandemic-related literature documents global decreases in patient volume across the trauma-care spectrum. Considerable reductions in ED trauma volumes were noted internationally, with rates of patients presenting to US EDs decreasing as much as 42% in general and 22% for musculoskeletal complaints [8-11]. Specific to orthopedic trauma, a systematic review of fractures during the pandemic demonstrates significantly decreased fracture rates compared to pre-pandemic levels; however, there is evidence suggesting that the only upper extremity fractures with significantly altered proportions during the pandemic were hand and wrist fractures, while proportions of clavicle, humerus, and forearm fractures remained stable [12]. Other studies note no change in rates of distal radius fractures during the pandemic but propose concern for increased fragility fractures due to reduced physical activity in elderly populations, increasing the risk of developing or worsening osteoporosis in the future [13]. While our results echo these trends, they appear to be filtered through the lens of patient sex: among male patients, rates of hand fractures decreased significantly and were surpassed by the rate of finger fractures that remained stable from prior years. Wrist fractures in female patients continued to be the most common fracture pattern both before and during the pandemic. The high rates of wrist fractures and upper arm fractures observed in female patients over 65 raises concern for osteoporosis-related fragility fractures; however, the downstream effects of the pandemic on bone health and the treatment and management of osteoporosis remain to be seen. 

The majority of non-sports-related products implicated in upper extremity fractures and dislocations are related to structural components of buildings and homes, such as floors, stairs, walls, beds, and doors. With high rates of wrist and upper arm fractures in female patients over 65, a substantial portion of injuries from structural components such as floors and stairs may represent fragility fractures or fractures secondary to accidental falls. The decreasing rates of upper extremity injuries from these fixtures during the pandemic may reflect a greater propensity for injury in non-home buildings that are less familiar to patients than a home environment, which many were confined to, particularly the elderly. As previously discussed, the effects of decreased activity on bone health and the risk for fragility fractures remain to be seen. Interestingly, injuries related to pet supplies and equipment increased substantially during the pandemic. While there is debate surrounding whether rates of pet ownership increased during the COVID-19 pandemic [14], individuals who spent more time in their places of residence likely were more exposed to both their pets and existing pet equipment in their homes, increasing their risk for injuries from these specific products more so than other immobile, structural fixtures in their homes.

Changes in injury trends during COVID-19 offer valuable insight into the evolution of athletic activities and sports during the pandemic. With many efforts to promote social distancing, including the suspension of organized sports, it is no surprise that sports-related mechanisms of injury exhibited sizeable decreases during the pandemic [15]. Reflecting existing consumer reports of biking popularity and corresponding bike production shortages, rates of bicycle-related injuries increased during the second year of the pandemic, with the incidence decreasing in 2020 before increasing in 2021 to higher than pre-COVID-19 rates. Rates of bicycle-related injuries are significantly divided by patient sex, with a much greater decrease in bike-related injuries observed among female patients as compared to male patients. On the contrary, skateboarding injuries similarly increased during the pandemic; however, rates of injuries among female patients increased over eight-fold while rates in male patients did not even double. Both biking and skateboarding reflect sports-related activities that can be performed individually and in an outdoor environment, which may explain their popularity in the setting of social isolation. Injuries from team sports such as basketball and soccer decreased among both male and female populations during the pandemic, with much larger decreases noted in female patient populations. Sports-related injuries in any extremity are estimated to result in fractures at a five-fold higher rate than dislocations [16]; subsequently, while the considerable decrease in sports-related upper extremity dislocations more so than fractures is surprising, it may be explained by injury patterns specific to upper extremities given that nearly two-thirds of sports-related dislocations involved the shoulder or finger. The shifting proportions of injuries occurring in sporting/recreational facilities and schools to homes and farms or ranches further align with these findings and offer further insight into reported shifts in injury locale from outdoor or public locations to homes [11,17]. 

The start of the COVID-19 pandemic certainly impacted patient disposition following presentation to the ED. The number of patients admitted in 2020 is substantially higher than in prior years, although admission rates decreased closer to pre-pandemic levels by 2021. When interpreted in the context of decreased rates of fractures and dislocations in 2020, increased admission rates may be explained by patients who tested positive for COVID-19 upon presentation or were admitted for requisite COVID testing prior to operative management. Alternatively, the same factors that Lim et al. [17] attribute to increased fracture-related mortality during COVID-19, such as delays in seeking medical care, may increase morbidity in general, further exacerbating increased admission rates. Decreased admission rates the following year may reflect a combination of lower COVID-19 transmission in 2021 in addition to hospital-specific protocols that are better adapted to managing resources and COVID testing capabilities. The considerable decrease in interhospital transfers in both 2020 and 2021 may likewise reflect prehospital and hospital system-based efforts to balance resources and limit interfacility transfers. Interestingly, the increase in patients leaving AMA in 2021 is also substantial, with an estimated 1.8 times higher rate in this year than in previous years, potentially indicating a reluctance to remain in the hospital for orthopedic injuries that culminated in 2021 after a year of the COVID-19 pandemic. The potential reluctance to remain in the ED for treatment may similarly explain the overall decrease in the number of patients presenting to the ED with orthopedic injuries in general, given that patients sustaining more minor injuries may have opted for telehealth visits or delayed treatment in order to avoid iatrogenic COVID-19 exposure. 

As with any database-driven study, limitations to our data are primarily the result of limitations of the NEISS database. Rates of fractures and dislocations in our studies are limited to injuries that presented to US EDs; subsequently, these rates may underestimate patients who did not receive medical attention for fractures to non-weight-bearing upper extremities or patients that presented to orthopedic or urgent care clinics either virtually or in-person. The societal effects of COVID-19 in the US occurred at different rates nationwide, so trends in the NEISS database may reflect pre-COVID-19 years in 2020 for different geographical regions. Additionally, data parameters were limited by what is reported in the NEISS database, as well as the potential for human error, given how NEISS data are collected by individuals at geographically and institutionally diverse hospitals across the US. Population estimates also introduce an additional challenge during the COVID-19 pandemic. Although NEISS data is intended to calculate population estimates based on census data, census projections were used for the 2020 and 2021 population data and, unfortunately, do not account for deaths due to COVID-19. Furthermore, the NEISS population weights may not be as generalizable to the nation as in typical years since COVID-19 influenced behaviors differently in the region for a variety of societal reasons as well as local case levels.

## Conclusions

The COVID-19 pandemic was associated with epidemiological and activity changes regarding upper extremity fractures and dislocations presenting to EDs. The present study demonstrates notable decreases in rates of upper extremity fractures and dislocations, increases in rates of injuries related to outdoor and individual sports such as skateboarding with corresponding decreases in rates of injuries related to organized sports such as basketball, increases in the rates of injuries occurring in homes and in association with pet supplies, and decreases in rates of injuries occurring in schools and places of recreation observed during the pandemic. Additionally, trends observed among patient disposition specific to the pandemic, such as increasing rates of patient admission, observation, and AMA departure with decreasing rates of discharge and transfer, offer insight into the burden of upper extremity injuries on the healthcare system during this critical time. While upper extremity orthopedic injuries remained common through the pandemic, the early pandemic was associated with higher rates of hospital admission that normalized closer to pre-pandemic levels by 2021, which may herald a shift and return to pre-pandemic trends. Future research will determine the long-term downstream effects of COVID-19 on activity-related orthopedic injuries and bone health.
